# Beyond the Scalpel: Advancing Strategic Approaches and Targeted Therapies in Nonexcisable Melanomas

**DOI:** 10.1155/2024/2167176

**Published:** 2024-08-27

**Authors:** Vivian Li, Kelly Frasier, Julia Vinagolu-Baur, Olivia Chapman, Alexandra Loperfito, Kathleen Daly, Viktoria Taranto

**Affiliations:** ^1^ Nuvance Health, Vassar Brothers Medical Center, Poughkeepsie, NY 12601, USA; ^2^ State University of New York, Upstate Medical University, Syracuse, NY 13210, USA; ^3^ Mercy Health St. Elizabeth Boardman Hospital, Youngstown, OH 44512, USA; ^4^ Edward Via College of Osteopathic Medicine, Blacksburg, VA 24060, USA; ^5^ The Medical College of Georgia at Augusta University, Augusta, GA 30912, USA; ^6^ New York Institute of Technology College of Osteopathic Medicine, Glean Head, NY 11545, USA

## Abstract

Melanoma in challenging anatomical locations such as the face, acral surfaces, and mucosal areas presents unique hurdles for surgical excision. This review examines alternative nonsurgical treatment modalities in the context of these complexities, addressing the gaps in current guidelines and the varied efficacy of existing therapies. A comprehensive literature search was conducted using PubMed, Embase, and Web of Science databases. The review focuses on peer-reviewed articles discussing nonsurgical treatment options for melanoma in complex anatomical locations. Articles were screened by three independent researchers, ensuring a broad analysis of topical agents, immunotherapies, radiotherapies, and targeted therapies. The review highlights significant advancements in localized treatments such as imiquimod and intralesional therapy with talimogene laherparepvec (T-VEC), which show promise in managing nonexcisable melanomas. BRAF and MEK inhibitors, as well as checkpoint inhibitors targeting CTLA-4 and PD-1/PD-L1 pathways, demonstrate improved survival rates but pose challenges with resistance and systemic side effects. Radiotherapy serves as an adjunctive strategy due to melanoma's inherent radioresistant properties. Despite advancements, there is a notable absence of comprehensive, evidence-based protocols to guide the treatment of melanoma in these critical areas. This paper underscores the need for standardized treatment guidelines that account for the efficacy, side effects, and psychosocial impacts of therapies. Future research should focus on refining existing treatments and exploring innovative modalities to enhance patient outcomes in the management of nonexcisable melanomas. Comprehensive guidelines and long-term efficacy studies are essential to optimize care and improve the quality of life for patients afflicted with melanoma in challenging anatomical locations.

## 1. Introduction

Anatomic locations such as the face, acral surfaces, and mucosal areas pose unique challenges in devising effective therapeutic strategies for melanoma excision, necessitating an exhaustive examination of alternative medical modalities. Melanomas of the head and neck account for nearly 20% of all malignant melanomas, with 65% of those presenting in the facial region [1]. Though common in frequency, these melanomas pose significant challenges in terms of treatment due to the intricate anatomy of the face. Other locations, including acral surfaces and the mucosa, are not nearly as common but pose even greater challenges due to the progressive stage at diagnosis [1]. Acral melanomas only make up 4–6% of all melanomas in Caucasians but are the most common subtype seen in Asians and African Americans [2]. Even rarer are mucosal melanomas which contribute to 0.8–3.7% of all melanoma cases in Caucasians [2]. Despite its infrequent occurrence, this anatomical location serves as one of the most aggressive melanomas with a significantly worse prognosis. Each of these locations varies in frequency among patients, but all pose unique challenges in terms of treatment decisions.

Current studies have begun to unravel promising avenues, exploring the application of topical agents, immunotherapies, and radiotherapies in addressing melanomas nestled within challenging anatomical contexts. However, despite the emergence of these alternative approaches, a critical gap persists in the lack of comprehensive guidelines and evidence-based protocols to systematically guide clinicians in managing nonexcisable melanomas. As such, this review seeks to critically analyze the existing literature, scrutinizing both the successes and limitations of these alternative modalities and expanding upon the associated challenges in refining and standardizing treatment approaches.

In envisioning the future trajectory of melanoma therapeutics, the discourse extends towards a proactive call for research endeavors aimed at refining existing modalities, evaluating their long-term efficacy, and discerning potential side effects. Furthermore, the exploration of innovative strategies, including targeted therapies and nanotechnology-based interventions, emerges as a focal point in this academic pursuit. This literature review is structured to present the current nonsurgical treatment modalities for nonexcisable melanomas, examine the anatomical challenges to surgical excision, identify existing limitations, and outline promising future research directions that aim to transform the nuances of melanoma therapeutics.

## 2. Literature Review

### 2.1. Materials and Methods

This narrative review was designed to comprehensively analyze and synthesize the current state of research concerning nonexcisable melanomas in anatomically challenging locations, highlighting alternative therapeutic strategies beyond surgical excision. The focus was primarily on evaluating nonsurgical interventions such as topical therapies, immunotherapies, radiotherapies, and emerging nanotechnology-based interventions, emphasizing their efficacy, limitations, and clinical outcomes. A literature search was formulated to gather peer-reviewed articles from PubMed, Embase, and Web of Science databases. Search terms used were “melanoma” and any of the following: “non-excisional,” “non-surgical,” “immunotherapy,” “radiotherapy,” “nanotechnology,” “anatomic challenge,” OR “topical.” The search strings were adapted for each database to enhance the efficiency and comprehensiveness of the search process. The results of the search were independently screened by three researchers to ensure a thorough and unbiased selection process. Data were synthesized qualitatively to highlight key findings, differences in treatment approaches, and consensus or discrepancies among the studies. The review particularly focused on the effectiveness of each treatment modality, challenges in treatments, and any reported long-term outcomes or adverse effects for nonexcisable melanomas.

### 2.2. Topical and Intralesional Therapy

#### 2.2.1. Imiquimod

Topical therapies offer a localized approach to melanoma treatment by targeting the immune system directly at the tumor site. Among topical agents, imiquimod has been the most extensively studied, though the evidence largely stems from case studies and a limited number of randomized clinical trials, with a notable absence of high-quality evidence supporting its use as a primary therapy in nonselected cases of melanoma in situ [3]. Imiquimod stimulates toll-like receptor 7 (TLR7) and triggers a cascade of cytokine releases, such as interferon-alpha [4]. This process initiates both innate and adaptive immune responses against tumor cells, showcasing its potential in combating melanoma. Despite the potential of topical treatments, the research landscape reveals a paucity of comprehensive data on their efficacy against the invasive nature of melanoma.

Nonetheless, practice guidelines have evolved to consider topical imiquimod as a viable alternative in specific scenarios where surgery or radiotherapy is not suitable [5]. A systematic review by Mora et al. highlighted that imiquimod treatment for lentigo maligna (LM), a subtype of melanoma, yielded histologic and clinical clearance rates of 76.2% and 78.3%, respectively, underscoring its potential in certain cases [6]. Supporting this, Tio et al. found that 84.2% of LM patients treated with imiquimod experienced complete clearance, reinforcing the therapeutic benefits of imiquimod [7].

Further evidence from two randomized control trials corroborates the efficacy of imiquimod in LM treatment, with one trial reporting a 64% complete response rate among patients and another observing a significant reduction in the overall LM score one month after treatment compared to controls [8, 9]. A prospective study illuminated the long-term outcomes of imiquimod treatment, reporting overall survival rates of 85.5% at five years and 70.4% at ten years for LM patients [10]. Moreover, the study identified the localization of LM in the nasal region as a prognostic factor for disease-free survival. These findings collectively highlight imiquimod's potential as a promising targeted therapy for nonexcisable melanoma, underscoring the need for more extensive research to conclusively validate its efficacy.

#### 2.2.2. Talimogene Laherparepvec (T-VEC)

Intralesional therapy with talimogene laherparepvec (T-VEC), an oncolytic herpes simplex virus (HSV) type 1 modified to produce granulocyte-macrophage colony-stimulating factor (GM-CSF), aims to specifically target melanoma cells while sparing healthy tissue [11]. This innovative treatment leverages T-VEC's dual-action mechanism: it directly causes the lysis of melanoma cells at the injection site and induces a systemic antitumor immune response by releasing viral antigens [12, 13].

Clinical trials have underscored T-VEC's efficacy, particularly in patients with stage IIIB, IIIC, or IVM1a melanoma and those who have not received prior treatment [11, 14]. These trials documented not only improved overall survival but also enhanced quality of life among participants, leading to the approval of T-VEC in 2015 in the United States for local management of unresectable cutaneous, subcutaneous, and nodal lesions after initial surgery and in Europe for similar indications in regionally or distantly metastatic melanoma (stages IIIB-IVM1a) [15, 16]. This treatment achieves disease control through both the locoregional lytic effects of the virus and its systemic immune-mediated actions, offering a strategy for managing melanoma in areas where surgical options are limited.

Importantly, T-VEC is capable of inducing responses in visceral lesions via systemic immune effects and has the potential for activating T-cells that target metastases in anatomically similar sites when injected into dermal, subcutaneous, and nodal metastases [17]. For melanomas located in the head and neck regions, where surgical intervention is not feasible, T-VEC monotherapy has demonstrated an impressive overall response rate (ORR) of 80% in a study by Franke et al. [18]. Further research indicates T-VEC's role not just in treating locoregionally advanced melanoma but also as an option for patients with stable or regressive systemic metastases who have developed resistance to immune checkpoint inhibitors or targeted therapies [19, 20].

### 2.3. Targeted Therapy

The introduction of BRAF inhibitors marked a significant advancement in the management of advanced melanoma, highlighting the critical role of the BRAF and MEK proteins in melanoma pathogenesis through their involvement in the mitogen-activated protein kinase (MAPK) pathway [21]. In approximately 50% of cutaneous melanomas, a mutation involving the substitution of valine with glutamic acid at position 600 (V600E) within the BRAF gene occurs, resulting in the uncontrolled proliferation and survival of melanoma cells [22, 23]. BRAF inhibitors, such as vemurafenib and dabrafenib, have demonstrated significant efficacy in treating patients with BRAF-mutant melanoma. In addition, MEK inhibitors such as trametinib and cobimetinib target downstream elements of the MAPK pathway, offering a broader impact by affecting both tumor and normal cells, unlike BRAF inhibitors which only target mutated tumor cells [24, 25].

However, the use of BRAF inhibitor monotherapy is often limited by the development of resistance within about seven months, leading to the strategic implementation of combination therapy [26]. Combining BRAF inhibitors with MEK inhibitors helps to block multiple downstream points, mitigating the risk of resistance and enhancing treatment efficacy [27]. Despite the benefits, the dual function of MEK inhibitors necessitates careful dosing to minimize toxicity risks, as they suppress function in normal cells as well [24]. Given these considerations, the National Comprehensive Cancer Network (NCCN) recommends combination therapy as a preferred regimen for treating unresectable BRAF V600E melanoma, underscoring the efficacy of these therapies while also acknowledging the potential for increased toxicity and the challenges associated with resistance [28].

### 2.4. Immunotherapy

The treatment landscape for melanoma has been revolutionized with the introduction of immunotherapies, particularly checkpoint inhibitors targeting CTLA-4 and PD-1/PD-L1 pathways. This shift from direct tumor targeting to immune system modulation allows for the recognition and destruction of melanoma cells, offering a nonsurgical approach for managing melanoma in locations where surgical intervention is not feasible or carries significant risk. The systemic nature of these therapies is especially advantageous for melanomas in anatomically challenging locations, providing an effective treatment option without the need for direct tumor access.

#### 2.4.1. Anti-CTLA-4 Agents

CTLA-4, exclusively expressed in T-cells, plays a critical role in regulating early T-cell activation. It acts by competing with CD28 for binding to B7 molecules, thereby inhibiting the costimulatory signal essential for T-cell proliferation, and contributes to the downregulation of T helper cells and the stimulation of regulatory T-cells, which have immunosuppressive functions [29, 30]. By blocking this checkpoint, anti-CTLA-4 therapies such as ipilimumab amplify T-cell activation and proliferation, significantly enhancing the immune system's ability to target and destroy melanoma cells. This blockade leads to an increased production of interleukin 2 (IL-2) and creates an environment conducive to immune-mediated tumor destruction [30, 31].

Ipilimumab, the first FDA-approved immune checkpoint inhibitor, has shown significant efficacy in treating unresectable stage III or stage IV melanoma. A phase III clinical trial revealed that ipilimumab extended median survival to 10.1 months compared to 6.4 months with gp100 [32]. In a separate study, it was shown that 20% of patients treated with ipilimumab survived at least two years following the conclusion of therapy, marking the first therapy to demonstrate a survival benefit in metastatic melanoma patients [33].

#### 2.4.2. Anti-PD-1/PDL-1 Agents

The PD-1 pathway plays a pivotal role in regulating T-cell activation through its ligands PD-L1 and PD-L2, inhibiting T-cell proliferation and IL-2 production, thereby reducing T-cell survival [30]. In contrast to CTLA-4's specific expression on T-cells, PD-1 is found on T-cells, B-cells, and myeloid cells; this pathway limits T-cell activity in peripheral tissues during inflammatory responses [29, 34]. Inhibiting the PD-1/PD-L1 pathway plays a vital role in reinstating the immune system's natural function, which activates cytotoxic T-cells and enhances their infiltration into the tumor microenvironment, resulting in the regression of melanoma [35, 36].

Several agents targeting the PD-1/PD-L1 pathway, including nivolumab, pembrolizumab, and atezolizumab, have been approved for melanoma treatment. Studies have shown pembrolizumab to achieve a 24-month survival rate of 55%, which is significantly higher than ipilimumab's 43% [37]. Moreover, nivolumab demonstrated a one-year overall survival rate of 72.9% in untreated melanoma patients, outperforming the 42.1% survival rate in the dacarbazine control group [38]. Notably, the CheckMate 067 trial marked a pivotal moment in the treatment of advanced melanoma, revealing a substantial increase in overall survival with the combined use of nivolumab and ipilimumab, in contrast to nivolumab and ipilimumab monotherapies [39].

According to the NCCN guidelines, the recommended initial approach for treating stage IB (T2a) or II (T2b or higher) melanoma involves wide excision followed by adjuvant therapy, with pembrolizumab or nivolumab being notable adjuvant options [28]. Based on clinical trial results, the guidelines highlight anti-PD-1 monotherapy with pembrolizumab or nivolumab, or combination therapy with nivolumab and ipilimumab, as preferred regimens with the highest level of evidence to support their efficacy.

### 2.5. Radiotherapy

Radiotherapy is a therapeutic approach that destroys cancer cell DNA leading to slowed growth or death of cancerous cells that the body can remove [40]. Various types of radiotherapy and dosages can be implemented depending on the etiology, disease advancement, patient, and location. On a broad scope, the type of radiation can be thought of as external radiation versus brachytherapy, with external radiation being the most commonly used in melanoma [41].

Although advances have been made in radiotherapy technology, its use in melanoma treatment is often limited by data suggesting melanoma's radioresistant properties [42]. Thus, its role as a definitive treatment option for melanoma is limited, and it is more often implemented as an adjunctive strategy. Definitive radiotherapy may be considered in cases of LM, desmoplastic melanoma limited by unobtainable surgical margins, difficult reresection, mucosal melanoma, ocular melanoma, and palliation [43, 44]. In LM, the typical radiotherapy dose is 50–56 Gy in 2 Gy fractions to a depth of 5 mm [45]. For unresectable head and neck mucosal melanomas, the use of definitive radiotherapy at 50–55 Gy has been reported in various case studies with the complete response obtained.

However, definitive radiotherapy is advantageous in that it can be utilized in managing melanomas located in areas where surgical access is challenging, thereby limiting the use of Mohs micrographic surgery or wide excision. This offers an alternative when the size of the tumor risks damage to vital structures [45]. The advantages of definitive radiotherapy are further understood when candidacy evaluation for surgery is investigated. Definitive radiotherapy is a viable option for patients who have ocular melanoma, are not good surgery candidates, or have medical interoperability [44]. This encompasses a range of comorbid medical conditions, such as diabetes mellitus, which complicates wound healing, as well as bleeding disorders or the use of blood-thinning medications that increase the risk of bleeding during surgery. In addition, age plays a significant role in these considerations.

Apart from the radioresistant nature of melanoma limiting the effectiveness of definitive radiotherapy, the adverse effects associated with radiotherapy also pose significant drawbacks. Radiotherapy is typically directed at the primary lesion site, yet it frequently results in damage to surrounding structures. Given that skin cancer often manifests in the head and neck regions, it poses a significant risk to the numerous critical structures located in these areas. A study by Souza et al. recognizes this risk by reporting damage to the arterial baroreceptors from radiation therapy causing or worsening hypertension [46]. Hypopigmentation from targeting melanocytes, sunburn-like reactions, hair loss, nausea, and fatigue may also result from radiotherapy, impacting the patient's psychosocial well-being and quality of life [41]. Considering the positive outcomes of the aforementioned nonsurgical treatments, it becomes evident that radiotherapy, immunotherapy, and topical and injected agents offer innovative treatment approaches and further evolution towards patient-centered regimens.

### 2.6. Anatomic Challenges to Surgical Excision

The surgical management of melanoma in anatomically sensitive areas demands a nuanced approach due to the inherent challenges these locations present. The NCCN recommendations for peripheral surgical margins based on tumor thickness are outlined in Table 1 [28]. However, they also acknowledge the necessity of modifying these margins to account for each patient's anatomical and functional considerations on a case-by-case basis. The complexity of treating melanoma in these regions lies not only in the surgical procedure itself but also in the broader implications for patient outcomes, including functional integrity, aesthetic considerations, and overall quality of life.

#### 2.6.1. Head and Neck

Head and neck melanoma treatment exemplifies the intricacies of balancing oncological efficacy with cosmetic outcomes, particularly in facial surgeries where the goal is to minimize disfigurement while ensuring thorough cancer removal. Adding to the complexity, the proximity of melanomas in the head and neck areas to vital structures such as nerves and blood vessels significantly complicates the surgical approach, and the extensive lymphatics of the head and neck result in the rapid progression of these tumors [47].

Carrera et al. presented a case illustrating these challenges, where initial attempts at wide-margin excision led to recurrence, rendering the patient a nonsurgical candidate [48]. The recurrence featured extensive invasion, including peri- and intraneural infiltration, affecting the infraorbital nerve canal and the surrounding soft tissue, and bone erosion. Treatment with ipilimumab in this patient led to the stabilization of the patient's stage IIIC melanoma without evidence of distant disease over 43 months of follow-up. Moreover, Buck et al. explored aesthetic outcomes from patients' perspectives following wide local excision in the head and neck areas, assessing appearance alteration, satisfaction, and emotional impact via a visual analogue scale (VAS) [49]. The findings indicated that skin grafts were associated with significantly less favorable VAS scores compared to other reconstructive options. Melanomas located on the eyelids and nose were associated with a greater emotional burden, reflecting the intricate challenges in these areas.

The management of melanoma on the nose and eyelids remains particularly challenging due to the anatomical complexities of the nasal pyramid and limited eyelid surface area, often resulting in narrower excision margins to spare functionality [47]. This decision, though at the cost of higher rates of treatment failure, ultimately improves the quality of life of the patient. In regions such as the perioral, perinasal, and periorbital areas, primary skin closure may lead to distortion and skin grafts for larger defects often result in suboptimal aesthetic outcomes due to mismatches in color and texture. In addition, flap failure due to ischemia, though rare, can lead to significant ocular complications [50].

#### 2.6.2. Acral Surfaces

Acral melanomas, found on the palms, soles, and under the nails, are not only notable for their distinctive location but also for their aggressive nature and tendency to be diagnosed at an advanced stage. The five-year survival rate for acral melanoma sits at 80.3% compared to the 91.3% seen in other cutaneous melanomas [2]. While the majority of acral melanomas are present on the feet, those on the hands tend to be thicker and more prone to ulceration [51]. Furthermore, a prospective cohort study over a 21-year period showed increased mortality in melanomas presenting on the nail [51]. In contrast, Wei et al. analyzed 1157 Chinese patients with acral melanoma and found that those with melanomas on the soles of their feet had worse prognoses than patients with primary lesions on the palms or under the nails [52].

The treatment of acral melanomas often requires either extensive plastic surgery reconstruction or amputation in severe cases [2]. In a retrospective study by Chakera and Thompson, it was found that distal amputation was necessary in 91% of the cases [53]. The decision to amputate carries profound implications for the patient. It has been proposed that wide local excision could be an alternative to amputation for low-risk primary lesions; however, this approach can risk incomplete tumor removal due to the nail bed's immediate proximity to the periosteum [54]. The postsurgical loss of mobility or sensation markedly affects the patient's quality of life, emphasizing the urgent need for adjunct or alternative therapies to surgery. In addition, longer recovery times prove increasingly detrimental to older patients, who may suffer from poor circulation in the hands and feet, further complicating their recovery and management of acral melanoma [2].

#### 2.6.3. Mucosal Sites

Mucosal melanomas, with their hidden presentation within the oral and nasal cavities, gastrointestinal tract, and genitourinary system, pose significant challenges in early detection and effective management, culminating in a five-year overall survival rate of only 14% [2]. The invasiveness required for surgical access in these regions often leads to considerable postoperative functional and aesthetic consequences. Specifically, treating mucosal melanomas in the craniofacial area frequently involves accessing the sinonasal tract and intradural space, where two important goals are achieving a complete tumor resection and meticulous closure of anterior skull base defects, which are crucial to prevent complications such as infections, cerebrospinal fluid leakage, and brain prolapse [55].

Mucosal melanomas tend to present in older populations, with more aggressive forms occurring in women, particularly impacting the genital tract [56]. Vulvovaginal melanomas, often found on the labia majora and the lower one-third of the anterior vaginal wall, present a surgical dilemma [57]. The spectrum of interventions ranges from conservative wide local excision to radical procedures such as vulvectomy/vaginectomy and pelvic exenteration [57]. The dilemma lies in the absence of a clear survival benefit and significant long-term consequences of radical surgery, such as morbidity, sexual dysfunction, and psychological distress. Concurrently, the absence of clear guidelines for conservative surgical margins leads to an elevated metastasis risk, exacerbated by the dense vascular and lymphatic network in the urogenital tract mucosa [57].

Similarly, in addressing localized anogenital melanoma, the debate between radical abdominoperineal resection and conservative wide local excision underscores the controversy in identifying the most effective surgical strategy [57]. Although abdominoperineal resection historically offered better local control, its effects on overall survival remain unclear. Thus, there has been a shift in emphasis towards achieving negative margins with conservative treatment while preserving sphincter function. Nevertheless, many patients ultimately develop metastatic disease despite locoregional control with surgery [57]. Given these complexities, mucosal melanomas frequently necessitate adjunctive neoadjuvant or adjuvant therapies, which introduce varying degrees of toxicity risks, particularly in older patients [2]. The high recurrence rate further emphasizes the need for innovative treatment modalities, such as targeted radiation and immunotherapy, to enhance patient outcomes.

#### 2.6.4. Evaluation and Summary of Existing Approaches

Strategic approaches and targeted therapies offer therapeutic variation in nonexcisable melanoma in anatomically challenging surgical locations. Advancements in these therapeutics, combined with a deeper understanding of their efficacy, have greatly improved both their application and effectiveness. Sas-Korczynska et al. analyzed cases of sinonasal mucosa melanoma treated with radiotherapy alone and reported complete remission and in some cases five-year disease-free survival [58]. This is very reassuring, especially in the case of location-limited mucosal melanoma, for the use of alternative therapy including definitive radiotherapy. However, larger studies are needed for broader evaluation. Further evidence for the use of radiotherapy in nonexcisional sinonasal melanoma is reported by Garousi et al. in which a total dose of 64 Gy in 32 fractions resulted in a decreased volume of the tumor and improvement in symptoms and signs with no progression at three months [59].

The use of alternative treatment in nonexcisable melanoma is further understood through implementation in LM and lentigo maligna melanoma (LMM). Hendrickx et al. performed a systematic literature review to examine the use of radiotherapy for LM and LMM and reported a recurrence rate of 0–31% with “good” to “excellent” cosmetic outcomes [60]. Furthermore, this study provided evidence that the recurrence rate is comparable to surgical resection for LM and LMM.

Support for the use of radiotherapy in palliation is further reinforced by Olivier et al., who demonstrated effective palliation of non-CNS metastasis from malignant melanoma, particularly with the use of higher-dose radiotherapy [61]. Notably, 9% of lesions had complete resolution of symptoms. Therefore, incorporating palliative care into treatment plans for melanoma, especially in cases of nonexcisable malignant melanoma, should be considered, as it often offers patients symptom relief, including pain management.

Topical therapies offer successful alternatives to surgical excision. Verga et al. report the case of a 77-year-old female with malleolar malignant melanoma treated with 5% topical imiquimod that resulted in complete resolution both histologically and clinically [62]. Immunotherapies have shown to be advantageous to nonexcisable melanoma through immune system regulation and providing treatment at a systemic level. Furthermore, Addeo et al. documented a case of metastatic malignant melanoma that was completely responsive to four cycles of ipilimumab, resulting in sustained remission in a 65-year-old male who had shown progression on primary treatment with the chemotherapy agent dacarbazine [63]. The outcomes of complete remission not only highlight the immune aspect of melanoma but also underscore the necessity for additional data and the broader application of immunotherapies, especially in cases of nonexcisable melanoma.

Currently, available treatment modalities vary in efficacy. Treatments may not only consist of the traditional modalities used for progressive cutaneous melanomas but may also involve the addition of other modalities based on melanoma location. In mucosal melanomas, where wide excision is difficult due to anatomical challenges, radiotherapy has shown success in tumor shrinkage. Similarly, in nonexcisable LMM, radiotherapy has been promising in terms of recurrence rate, with results that resemble excisable melanomas. Though the locations differ, radiotherapy's success opens discussions for its use in other nonexcisable melanomas. Furthermore, the use of topical therapies offers a second approach in these challenging melanoma cases.

The promising results of complete remission in progressive cases of metastatic melanoma warrant further exploration into the use of systemic therapy for nonexcisable melanoma. Several retrospective studies and prior clinical trials have shown improved survival in acral melanoma with the usage of immune checkpoint inhibitors such as pembrolizumab [2]. While there is an overall improvement in survival rates, it is important to consider the slightly reduced survival observed in Asian populations [2]. In those with mucosal melanomas, studies have indicated the increased clinical benefit of combined immunotherapies, such as the ipilimumab and nivolumab combination [2]. Treatment decisions ultimately are patient-driven, as many neoadjuvant and adjuvant therapies present toxicities.

### 2.7. Limitations and Future Research

Our comprehensive review of existing literature highlights a pressing concern; despite advancements in the treatment of nonexcisable skin cancers such as melanoma, there is a scarcity of guidelines and protocols on best practices for treatment options. As significant strides are made in treating nonexcisable melanomas and other skin cancers, our review highlights the imperative of developing comprehensive guidelines to ensure optimal patient treatment.

Consequently, the absence of centralized guidelines for treating nonexcisable melanomas imposes constraints on the current management of such cases. Certain therapies may exhibit greater potential for enhancing prognoses, while others might offer more preventive advantages, and the different intended purposes and evidence-based outcomes of certain therapies ought to be considered when healthcare professionals are deliberating between varying nonsurgical therapies.

A notable gap exists in the scientific literature regarding the long-term efficacy of treatments for nonexcisable melanomas. There is a pressing need for comprehensive, long-term studies to ascertain the efficacy of therapeutic interventions. This is imperative, as insufficient data hamper clinicians' ability to make informed treatment decisions for patients, especially in cases of nonexcisable melanomas where it is more challenging to monitor cancer growth and intervene to slow progression. Expanding research efforts, particularly through longitudinal investigations, is imperative to enhance our understanding and optimize the management of these challenging malignancies.

An encompassing understanding of potential long-term side effects across diverse skin cancer and melanoma therapies, spanning topical and intralesional treatments, targeted therapies, immunotherapies, radiotherapy, and surgery, is essential for informed clinical decision-making. Topical and intralesional therapies, often utilized for superficial lesions, may result in adverse effects such as skin irritation, hyperpigmentation, and scarring. Targeted therapies, while effective against specific molecular targets, can lead to systemic issues including fatigue, nausea, and liver toxicity. Immunotherapies, lauded for their ability to engage the immune system against cancer, may induce autoimmune reactions and endocrine dysfunction. Radiotherapy, a cornerstone in cancer treatment, carries risks of radiation dermatitis, fibrosis, and secondary malignancies. Surgical interventions, although crucial for tumor removal, may entail complications such as scarring, nerve damage, and lymphedema. An awareness of these potential long-term effects is vital for holistic patient care and the optimization of treatment strategies.

Furthermore, it is crucial to acknowledge the enduring psychosocial ramifications of the dermatological side effects resulting from cancer therapy. A recent systematic review conducted by Almeida et al. underscores this concern, highlighting that cancer treatments cause the most varied skin changes compared to the treatment of other diseases. As a result, this leads to diminished self-esteem, reduced quality of life, and distorted body image. Consequently, these factors often contribute to heightened levels of stress, anxiety, and depression among affected individuals [64].

As emphasized in this exhaustive review of the medical literature, targeted therapies have emerged as promising interventions for the treatment of nonexcisable melanomas, offering a tailored approach to combating these challenging malignancies. Unlike conventional chemotherapy, which often lacks specificity and can lead to widespread toxicity, targeted therapies selectively inhibit molecular pathways implicated in melanoma progression. By targeting specific genetic mutations or aberrant signaling pathways driving tumor growth, these therapies hold the potential to achieve more precise and effective tumor control. Despite these promising attributes, challenges such as the development of resistance mechanisms and off-target effects underscore the need for ongoing research to optimize treatment strategies and enhance long-term outcomes [65].

#### 2.7.1. Future Directions

The advent of novel, nanotechnology-based approaches has been pivotal in advancing the treatment of melanoma. Nanoscale drug delivery systems offer multiple advantages including enhanced drug solubility, improved drug stability, prolonged half-life, optimized bioavailability, targeted tumor delivery, and minimized side effects [66]. In addition to targeted therapies, nanoscale liposomes have shown to be promising; Bedikian et al. found that liposomes have the potential to significantly increase the half-life of drugs in circulation and can even enhance drug efficacy in the treatment of melanoma, particularly in cases such as where drugs target the cell cycle [67].

Future research should prioritize the development of comprehensive guidelines and evidence-based protocols for standardized treatment of melanomas in challenging anatomical sites. The current landscape of nonexcisional treatments, such as immunotherapy and targeted topical and intralesional therapies, has shown promise, but a lack of standardized protocols hampers their widespread implementation. The development and implementation of standardized guidelines would both ensure consistency and optimize treatment outcomes. Standardized protocols should be evidence-based, considering long-term outcomes, recurrence rates, and side effects of various treatment modalities in nonexcisional melanomas classified by location. Though melanomas are currently classified by growth pattern and tumor stage, future innovative research may incorporate a classification system based on specific anatomical locations. This will enable the assessment of nonexcisional treatment modalities by their efficacy in various anatomical locations and aid in guiding personalized care for individual patient needs.

Furthermore, there exists a pressing need to identify areas for refinement within existing treatment modalities to enhance both efficacy and patient tolerability. Innovative targeted treatment modalities and nanotechnology approaches show promise in reducing mortality and improving outcomes for nonexcisional melanoma treatment, and research efforts should strive to refine these treatments. With goals of implementing standardized protocols and refining existing treatment approaches, future research can pave the way for more personalized and effective nonexcisional melanoma treatments.

## 3. Conclusions

Melanoma located in complex anatomical areas such as the head, neck, acral, and mucosal surfaces presents a significant challenge due to the impracticality of traditional wide excision. This review highlights important advances and reveals critical gaps in the treatment of nonexcisable melanomas. Standardized, evidence-based guidelines are needed to optimize therapeutic outcomes by balancing efficacy and side effects and by considering the psychosocial impacts on patients. Emerging therapies such as targeted topical agents, immunotherapies, radiotherapies, and nanotechnology-based interventions have shown promise in managing these challenging cases. Future research should focus on assessing the long-term efficacy of these treatments, refining therapeutic protocols, and exploring innovative treatment options. Enhanced focus on these areas will be the key to improving care and enhancing the overall outcomes for patients with nonexcisable melanomas.

## Figures and Tables

**Table 1:** Wide excision surgical margins for primary cutaneous melanoma.

Tumor thickness	Recommended peripheral surgical margins (cm)
In situ	0.5–1
≤1.0 mm	1
>1.0–2 mm	1-2
>2.0–4.0 mm	2
>4.0 mm	2

## Data Availability

Data sharing is not applicable to this article as no datasets were generated or analyzed during the current study.
